# Developing and implementing an accreditation system for health promoting schools in Northern India: a cross-sectional study

**DOI:** 10.1186/1471-2458-14-1314

**Published:** 2014-12-22

**Authors:** Jarnail Singh Thakur, Deepak Sharma, Nidhi Jaswal, Bhavneet Bharti, Ashoo Grover, Paramjyoti Thind

**Affiliations:** School of Public Health, RN Dogra Block, Post Graduate Institute of Medical Education and Research, Sector-12, Chandigarh, 160012 India; Advanced Paediatric Center, Postgraduate Institute of Medical Education and Research, Chandigarh, India; Indian Council of Medical Research (ICMR), New Delhi, India; School Health Programme, Chandigarh Administration, Chandigarh, India

**Keywords:** Accreditation, Health promoting schools, Manual

## Abstract

**Background:**

The “Health Promoting School” (HPS) is a holistic and comprehensive approach to integrating health promotion within the community. At the time of conducting this study, there was no organized accreditation system for HPS in India. We therefore developed an accreditation system for HPSs using support from key stakeholders and implemented this system in HPS in Chandigarhterritory, India.

**Methods:**

A desk review was undertaken to review HPS accreditation processes used in other countries. An HPS accreditation manual was drafted after discussions with key stakeholders. Seventeen schools (eight government and nine private) were included in the study. A workshop was held with school principals and teachers and other key stakeholders, during which parameters, domains and an accreditation checklist were discussed and finalized. The process of accreditation of these 17 schools was initiated in 2011 according to the accreditation manual. HPSs were encouraged to undertake activities to increase their accreditation grade and were reassessed in 2013 to monitor progress. Each school was graded on the basis of the accreditation scores obtained.

**Results:**

The accreditation manual featured an accreditation checklist, with parameters, scores and domains. It categorized accreditation into four levels: bronze, silver, gold and platinum (each level having its own specific criteria and mandate). In 2011, more than half (52.9%) of the schools belonged to the bronze level and only 23.5% were at the gold level. Improvements were observed upon reassessment after 2 years (2013), with 76.4% of schools at the gold level and only 11.8% at bronze.

**Conclusions:**

The HPS accreditation system is feasible in school settings and was well implemented in the schools of Chandigarh. Improvements in accreditation scores between 2011 and 2013 suggest that the system may be effective in increasing levels of health promotion in communities.

## Background

The “Health Promoting School” (HPS) is a holistic and comprehensive approach to integrate health promotion within the community. It can provide an ideal setting to enhance both health and learning. It can help prevent poor dietary habits and substance abuse and can improve school performance [1, 2]. It can decrease the risk of certain problems such as eating disorders, obesity, cardiovascular disease and cancer [3, 4]. In India, the Right to Education Act makes education a fundamental right of every child between the ages of 6 and 14 years [5]. These are the formative years of a child’s development. The interaction between schoolteachers and students provides a unique opportunity for health promotion that can be sustained and reinforced over time. It is an internationally recognized fact that school is an appropriate setting to improve youth health.

The World Health Organization (WHO) Global School Health Initiative was launched in 1995 to mobilize and strengthen health promotion and education activities at the local, regional, national and global levels [6]. The goal was to increase the number of schools that can be defined as HPSs. Countries including Sri Lanka, Bangladesh and Malaysia are implementing certain components of the School Health Program [7]. Studies have indicated that the implementation of the HPS program has had a positive impact on students' health behaviors [8, 9].

In many countries around the world, earmarked taxes on tobacco and alcohol have been used to raise funds for health promotion. The Thai Health Promotion Foundation (ThaiHealth) is one such model that has been widely regarded as a national innovative financing mechanism for health, in which a 2% surcharge tax has been levied on tobacco and alcohol products (Thai Health Promotion Act). Under the umbrella of “Healthy Thailand”, the HPS program in Thailand has been successfully managed and expanded. Both public and private schools participate in this. In each school, a team consisting of teachers, students, parents, health personnel and community representatives develop a plan of action to achieve HPS status. In addition, health promotion is integrated into the school curricula and textbooks [10].

Accreditation is a public recognition of the achievement of required standards by an organization. It is demonstrated through an independent assessment of that organization’s level of performance in relation to the standards. In India, the Quality Council of India (QCI) has developed an accreditation system for school governance [11]. However, there is no organized accreditation system for HPS in India. We therefore developed an accreditation system for HPS with support from key stakeholders and implemented accreditation for HPSs in Chandigarh territory, India.

## Methods

### Development phase

A quasi-experimental pre-intervention and post-intervention study was undertaken over a period of 2 years (2011–2013). The School Health Program Team of Chandigarh organized a sensitization meeting with the public (government) and private schools in Chandigarh. A meeting was also held with the education department of Chandigarh local government (Chandigarh Administration). Based on these groups’ inputs, the schools that volunteered to participate in the development of an HPS accreditation scheme were included in the study. Seventeen schools from Chandigarh (eight government and nine private) were selected for inclusion in the study, after gaining their informed consent. Demographic characteristics of the schools are shown in Table 1.

**Table 1:** **Demographic characteristics of schools that participated in the study**

S.No.	Type of school	No. of teachers	No. of students	Student Teacher Ratio	Physical education teachers	Counselors	Doctor	Dietician	Govt. assistance
1	Guru Gobind Singh SSS, Sec 35- B (P)	50	2154	1:43	3	1	1	1	Partial (95%)
2	Govt. Model SSS, Sector 10 (G)	60	2350	1:39	4	1	0	0	100%
3	Sacred Heart Sr. Sec. School, Sec 26 (P)	96	3166	1:32	4	2	0	0	Nil
4	St. John’s High School, Sec. 26 (P)	74	2008	1:27	3	3	1	0	Nil
5	DAV Model Sec 8 (P)	69	960	1:13	4	1	0	0	Partial (95%)
6	Jawahar NavodayaVidyalaya, Sec 25 (G)	31	508	1:16	2	0	1	1	100%
7	Govt. Model Sr. Sec. School, Sec 35 (G)	89	1629	1:18	3	0	0	2	100%
8	Guru Harkrishan Model Sr. Sec. Sec 38 (P)	50	954	1:19	2	1	1	1	Nil
9	Shishu Niketan, Sec. 22 (P)	83	3007	1:36	4	0	0	0	Nil
10	Govt. Model Sr. Sec. School, Sec 16 (G)	79	2393	1:30	4	0	0	0	100%
11	Govt. Model Sr. Sec. School, Sec 27 (G)	51	995	1:20	2	0	0	0	100%
12	Govt. Model Sr. Sec. School, Sec 37 (G)	78	2530	1:32	4	0	1	0	100%
13	Govt. Sr. Sec. School, Vill. Karsan (G)	30	2363	1:78	2	0	0	0	100%
14	St. Kabir School, Sec 26 (P)	69	1100	1:16	4	1	1	0	Nil
15	Bhawan Vidyalaya (P)	60	2080	1:34	3	1	1	0	Nil
16	Govt. Model Sr. Sec. Kaimbwala (G)	23	1143	1:50	1	0	0	0	100%
17	Carmel Convent School (P)	64	1443	1:23	3	1	1	0	Nil

Total of 17 schools (8 government and 9 private) participated in the study. The characteristic of each school in terms of number of teachers, students, and student-teacher ratio, availability of counselor, doctor and dietician and provision of financial assistance by the government has been stated in the table.

Numbers of teachers and students in government and private schools were similar (teacher-student ratios 1:33 and 1:27, respectively). However, there was only one counselor among the government schools compared with 11 counselors among the private schools.

A desk review on the HPS framework and school accreditation studies for a reference period of 10 years (2001–2010) was conducted to review HPS accreditation processes used in other countries. In addition to a detailed documentary review, an extensive literature search using Pubmed was undertaken. These reviews focused on health promoting schools and accreditation programmes for such schools. Discussions were held with key stakeholders on how best to develop a relevant, adaptable and achievable accreditation system (Table 2), specific to the context of Chandigarh and amenable to future scale up to other states in India. Special emphasis was given to developing skills pertaining to self-awareness, problem-solving, decision-making and interpersonal relationships.

**Table 2:** **Milestones of development and implementation of accreditation in Chandigarh**

S.No.	Time period	Key activities
1	August, 2008	Stakeholders’ workshop for Health Promoting Schools (HPS) was held at Chandigarh with following objectives:
		• Sensitization
		• Brainstorming
		• Discussion on HPS concept
2	October, 2009	A one day Workshop on HPS by School Health Programme, Chandigarh Administration to
		• Introduce the concept of Health Promotion in the schools.
		• 38 participants from 30 schools including District Education Officer (DEO) participated.
		• Lectures and group discussions were conducted.
3	August, 2010	A meeting was organized by Director Health Services, Chandigarh with key stakeholders including education department to initiate the process to select the schools on pilot basis for development of accreditation process.
4	October, 2010	A meeting of the representatives of Health department, investigators and Quality Council of India was held to discuss the development of draft accreditation process of schools.
5	Phase I: Development of Accreditation (April, 2011- May, 2012)	‘Development of a model for accreditation of school as Health Promoting schools in Union Territory, Chandigarh’ was supported by WHO Country Office for India and its implementation was initiated.
		• A workshop on Health Promoting Schools was organized by School Health Programme, Chandigarh Administration
		-Representatives from 17 willing schools (8 govt. and 9 private) participated.
		-Draft manual of Accreditation was discussed and finalized.
		• Baseline information on health promoting schools was collected from the participating schools.
		• 15 follow-up and reinforcement meetings with participating schools were organized by School Health Program, Chandigarh Administration.
		• Based on inputs and experience from schools, a manual of accreditation for Health Promoting Schools was finalized.
		• The manual of accreditation for Health Promoting Schools (HPS) was released by Governor of Punjab.
		• Key partners’ meeting from Education, Health and Finance department, Chandigarh Administration; Quality Council of India and School Health Program was held for implementation of accreditation.
6	Phase 2: Implementation of accreditation (2012–2013)	• The implementation started in 17 schools volunteered for implementation.
		• The schools made their plan of action, implemented and monitored with regular self-appraisal.
		• Technical support by School Health Program and investigators, on periodic basis.
		• A post-assessment in 17 schools was done.
		• The pre-post assessment in schools was analyzed and compared.

The accreditation of schools was not a one-time activity. Its development and implementation comprised of several milestones. The process was initiated in the year 2008, underwent number of expert consultation, meetings and workshops. Finally, a Manual on Health Promoting Schools was developed which described the procedure of accreditation including the checklist and scoring of accreditation. This accreditation process was implemented in the 17 schools on the pilot basis.

Based on the literature review and stakeholder discussion, a draft manual on HPS was developed that focused on grading schools into different HPS categories. A 1-day workshop was held in 2011 at Chandigarh with key stakeholders including representatives of the 17 schools, the Ministry of Health and Family Welfare (MOHFW), the Indian Council of Medical Research (ICMR), the Quality Council of India (QCI) and the WHO. During the workshop, participants discussed the HPS concept, grading criteria, checklist and manual draft. They were instructed to prepare their modalities and delivery of school health-related interventions such as “School Health Environment”, “School Health Services” and “The School Nutrition Program”. A second meeting was held to generate parameters specific to each of the eight selected domains, i.e., healthy school environment; presence and awareness of health promoting schools; school health services; school nutrition services; physical education; school counseling, psychological and social services; community leadership; and school involvement in HPS and its accreditation. The tool (checklist) was pilot tested in eight schools. The individual score in each of the eight domains was calculated for each participating school and the raw data was entered into SPSS version 16.0. Reliability analysis was done to check the reliability of the checklist. Fifteen follow-up and reinforcement meetings were organized by the School Health Program, Chandigarh Administration with participating schools. Based on inputs from schools and experts, a manual of accreditation for Health Promoting Schools was finalized.

### Implementation phase

During the 2011 HPS workshop, the pre-implementation assessment of the health promotion activities undertaken by the 17 participating schools was completed. Workshop participants were provided with a WHO manual of HPS and the 17 schools conducted activities to improve their HPS category over the subsequent 2 years. The School Health Department, along with collaborating institutes such as PGIMER and QCI, provided technical support and mentoring to the schools for self-improvement. The participating schools constituted the “Health Promotion Committee” with a specific mandate. They organized health promotion activities, and some of the schools focused on thematic health areas during certain periods. Details of all health-promoting activities were documented. Periodic self-assessment and follow-up activities were undertaken by the schools that participated in the workshop. Schools were routinely visited and guided by the Chandigarh School Health Program Officer and technical support was provided by the study investigators.

Post-implementation assessment of schools was carried out in 2013 for reassessment using the same accreditation checklist. The results of pre- and post-implementation assessments were analyzed and compared. Ethical clearance was obtained from the Institutional Ethics Committee of the Postgraduate Institute of Medical Education and Research, Chandigarh.

## Results

The results have been categorized into two phases: Development and Implementation phases.

### Development phase

**Figure 1:**
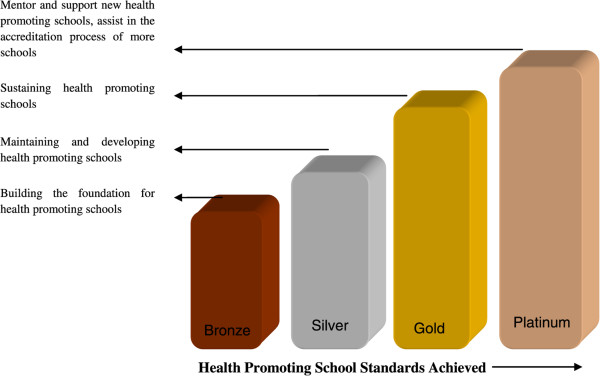
**Categories of Health Promoting Schools (HPS).** As given in the Manual of Health Promoting Schools, there are four levels of accreditation i.e. Bronze, silver, gold and platinum. Each level has its own standard and parameters. The school has to achieve those standards to reach to specific level. Bronze standard builds the foundation for HPS, silver level stands for maintaining and developing HPS, gold standard sustains the HPS and schools in platinum level mentor and support new HPS.

Qualitative analysis of the stakeholders’ meeting outcome generated 26 parameters for the accreditation checklist. A scoring system including all 26 parameters was developed (with 1 to 3 points awarded for each item) and scoring was undertaken. Cut-off for scores of 100–120, 121–150, 151–200 and >200 points were categorized as bronze, silver, gold and platinum levels, respectively (Figure 1). The 26 parameters were classified into eight specific domains. The reliability and validity of the checklist was established before it was finalized. The tool was pilot tested in eight schools. The reliability score of the checklist was 0.7.

The finalized accreditation levels are defined as follows:

Bronze level is the foundation level for HPS. At this level of accreditation, school administration understands and prepares to implement the HPS framework. They must develop a 1-year, need-based action and evaluation plan. School health committees are formed. Health cards, including immunization records for every student and records for students with special medical needs, must be implemented.

At the silver level, school management must maintain the requirements of the HPS bronze level. A need-based annual action and evaluation plan is implemented and monitored and the HPS must demonstrate improvements, along with increased involvement of parents and students.

At the gold level, in addition to maintaining the silver standard, schools must demonstrate further improvements. There must be promotion of environmental health and staff health and well-being. Wider community engagement must be strengthened and an induction package must be implemented that supports student health and well-being.

At the platinum level, a 3-year strategy to further improve school community well-being must be developed. A platinum HPS should mentor and support new HPSs (as a mother school) and assist in the accreditation process of more schools. As with silver and gold levels, annual action plans must be implemented and monitored.

### Implementation phase

Intervention for HPSs started in March 2011 after baseline assessment. The results of the pilot accreditation in 2011 and the reassessment conducted in 2013 are shown in Table 3. Nearly half (41%) of schools demonstrated an improvement in the provision of clean drinking water, clean toilets and adequate lighting for students between 2011 and 2013. The proportion of pilot schools with a waste management system increased from 18% (n = 3) in 2011 to 88% (n = 15) in 2013. The presence of a School Health Committee (SHC) was found in 53% (n = 9) of schools in 2013, compared with 18% (n = 3) of schools in 2011. Training for the HPS program in schools increased from 18% (n = 3) in 2011 to 71% (n = 12) in 2013. More than two-thirds (65%, n = 11) of schools were found to have a School Health Coordinator in 2013, responsible for carrying out overall health activities, compared with 12% (n = 2) of schools in 2011.

**Table 3:** **Comparison of schools after implementation of accreditation manual in 2011 and 2013**

S. No.	Parameter/Indicator	Best score (3)	
		2011	2013
		No. of schools (N = 17)	No. of schools (N = 17)
**I.**	**Healthy School Environment**		
1.	Access to adequate lighting, clean drinking water and clean toilets	3 (17.7%)	10 (58.8%)
2.	Sufficient dustbins for refuse disposal.	3 (17.7%)	15 (88.2%)
3.	School Safety and presence of evacuation plan for which everyone is trained. (Record and Observation)	2 (11.8%)	7 (41.2%)
**II.**	**Presence and awareness about Health Promoting Schools in schools**		
1.	Presence of school health committee	3 (17.7%)	9 (52.9%)
2.	Presence of a notice board	2 (11.8%)	11 (64.7%)
3.	Presence of posters and/or other means of publicizing and popularizing Health Promoting Schools in the school and local community.	3 (17.7%)	14 (82.4%)
4.	Student awareness and understanding of Health Promoting Schools concept, objectives and strategies.	3 (17.7%)	11 (64.7%)
5.	Training for Health Promoting Schools Programme.	3 (17.7%)	12 (70.6%)
6.	Presence of a coordinator for the Health Promoting Schools Programme.	2 (11.8%)	11 (64.7%)
7.	Curriculum which emphasizes on health subjects.	3 (17.7%)	13 (76.5%)
8.	Presence of sources and/or lectures on priority health subjects for students and staff.	3 (17.7%)	13 (76.5%)
9.	Staff is setting the role model.	1 (5.9%)	7 (41.2%)
**III.**	**School Health Services**		
1.	Presence of a health cards.	7 (41.2%)	15 (88.2%)
2.	Presence of first-aid kit.	4 (23.5%)	15 (88.2%)
3.	Training of students and staff on first aid.	4 (23.5%)	14 (82.4%)
**IV.**	**School Nutrition Services**		
1.	Nutrition education in school.	4 (23.5%)	13 (76.5%)
2.	Monitoring canteens/meals in the schools.	4 (23.5%)	13 (76.5%)
3.	Option of healthy food and drinks.	2 (11.8%)	14 (82.4%)
**V.**	**Physical Education**		
1.	A minimum number of hours of physical activity per week to all students in or outside the school curriculum.	4 (23.5%)	14 (82.4%)
**VI.**	**School counseling, psychological and social services**		
1.	Presence of social programmes and controlling health risk behavior.	3 (17.7%)	12 (70.6%)
2.	Adolescent Education Programme - life skill education.	2 (11.8%)	14 (82.4%)
**VII.**	**Community Partnership**		
1.	Community partners in decision-making and planning in the Health Promoting activities of the school.	3 (17.7%)	8 (47.1%)
**VIII.**	**Involvement of schools in establishing more Health Promoting Schools and their accreditation**		
1.	Mentor and support new Health Promoting Schools	1 (5.9%)	2 (11.8%)
2.	Assist in the accreditation.	0	0

The 17 schools have been accredited on eight specific domains pertaining to healthy school environment, Presence and awareness about Health Promoting Schools, school health services, school nutrition services, physical education, school counseling, psychological and social services, community participation, involvement of schools in establishing more health promoting schools and their accreditation. Intervention for Health Promoting Schools (HPS) was started in March 2011 after baseline assessment of 17 schools on eight domains. The post-intervention reassessment of the schools conducted in 2013.

The proportion of schools providing health cards to all students increased from 41% (n = 7) in 2011 to 88% (n = 15) in 2013. The proportion of schools possessing a first aid kit increased from 23% (n = 4) in 2011 to 88% (n = 15) in 2013. The majority of schools (82%, n = 14) had organized training of students and teaching staff on administration of first aid.

Regular monitoring of meals and canteens in schools was carried out. Fourteen (82%) of the schools included canteen facilities. Free midday meals were provided in all government schools (but not in private schools) and were monitored regularly. One private school had an independent canteen monitoring committee that regularly monitored the canteen menu, types of food and drinks being sold and hygiene and safety of the canteen. All schools included in the study were found to have appropriate playground facilities and 82% (n = 14) of them had designated hours each week assigned for physical activity as per the Central Board for Secondary Education (CBSE) norms in 2013 which include a minimum of five periods a week for physical activity [12], compared with 23% (n = 4) in 2011. The provision of life skills education in schools increased from 12% (n = 2) in 2011 to 82% (n = 14) in 2013.

During pre-intervention evaluation in 2011, only 25% (n = 2) of the government and 22% (n = 2) of the private schools belonged to the gold level of accreditation. However, post-intervention evaluation in 2013 showed that the proportion had increased to 62% (n = 5) and 89% (n = 8) in government and private schools, respectively. Overall, the proportion of schools at the gold level increased from 23% (n = 4) in 2011 to 76% (n = 13) in 2013 (Table 4). Therefore the accreditation system proved beneficial in terms of improving health promotion in the school setting.

**Table 4:** **Comparison of accreditation status of government and private schools in Chandigarh, 2011–2013**

Accreditation category	Type of schools								
	Government schools (N = 8)			No. of private schools (N = 9)			Overall		
	2011	2013	% age difference	2011	2013	% age difference	2011	2013	% age difference
Bronze	4 (50)	2 (25)	-25.0	5 (55.6)	0	-55.6	9 (52.9)	2 (11.8)	-41.2
Silver	2 (25)	1 (12.5)	-12.50	2 (22.2)	0	-22.20	4 (23.5)	1 (5.9)	-17.6
Gold	2 (25)	5 (62.5)	+37.50	2 (22.2)	8 (88.8)	+66.60	4 (23.5)	13 (76.4)	+52.9
Platinum	0	0	0	0	1 (11.2)	11.2	0	1 (5.9)	+5.9

**Note:** *Figures in parenthesis are percentages.*

Based on the total scores obtained by each school on the accreditation checklist, each school was categorized into platinum, gold, silver and bronze category. During pre-intervention evaluation in 2011, only 2 (25%) of the government and 2 (22%) of the private schools belonged to the gold level of accreditation. However, this proportion increased to 5 (62%) and 8 (89%), respectively after intervention in 2013. Overall, the proportion of schools at the gold level increased from 4 (23%) in 2011 to 13 (76%) in 2013.

## Discussion

The accreditation system developed by the experts and key stakeholders in Chandigarh is a practical tool to implement HPSs in the Indian context, and is the first of its kind in the country. The accreditation scheme follows a process of continuous improvement. Only Thailand in South East Asia has implemented such an accreditation system for schools. Once a particular level is reached, sustainability and aspiration to move to the next level is the goal. For sustainability, continuous active commitment and demonstrable support by governments to the ongoing implementation is required. There is a need to promote this strategy through a formal, written partnership between health and education ministries, to formalize commitment to this endeavor.

In Chandigarh, the accreditation system was piloted in 17 schools in 2011. The results have been quite encouraging. Both government and private schools benefited from the intervention (Tables 3 and 4). The proportion of schools at the gold level increased from 23% (n = 4) in 2011 to 76% (n = 13) in 2013.

The findings from many of the school-based interventions have validated their effectiveness in promoting the health of school children. A randomized controlled study was carried out in Sydney, Australia whereby the intervention schools were offered seminars and training in the HPS concept, encouraged to use a resource kit to help them establish their school as health promoting and invited to participate in a support network [13]. Pre- and post-measures of awareness, school structures and policies and practices to support the development of a HPS were taken and intervention and control schools compared. There was an increased level of awareness of the HPS concept among intervention schools. However, there were no significant changes in health-related policies and practices at the school level, among both intervention and control schools [13].

A case study in Hong Kong found that students in schools that had adopted the HPS framework had a more positive health behavior profile than those in non-HPS schools [9]. Some differences were found to be more significant among primary school students than secondary school students. This illustrates that early intervention for lifestyle changes is more effective. A systematic review was carried out to study the effectiveness of mental health promotion interventions for young people in low and middle income countries (LMICs) [14]. Fourteen studies of school-based interventions implemented in eight LMICs were reviewed. Mental Health Interventions for children indicated significant positive effects (improved self-esteem and coping skills) on students’ emotional and behavioral well-being [14]. Another review evaluating nine studies of HPS provided evidence that HPS has some influence on various domains of health for the school community, and there is scope for integrating health promotion into school policies and the curriculum [15]. A multi-component model of nutrition and lifestyle education was found to be effective and successful in improving the nutrition-related knowledge, eating habits and lifestyle practices of students, and resulted in beneficial changes in anthropometric and biochemical profiles of the Asian Indian adolescents [16]. Chandigarh is a Union Territory of India that is administered directly by the Central Government. There is no significant difference between government and private schools in terms of infrastructure. The teachers are well qualified and the student-teacher ratio is similar across school types. However, the socioeconomic profile of students differs between private convent schools and government schools, with children from high socioeconomic strata more likely to study in private convent schools. The accreditation of schools covered both government (non-affluent) and private (affluent) schools. The eight domains in the accreditation checklist were devised in consultation with representatives from both government and private schools, thereby reducing the possibility of bias towards a specific category of schools. Furthermore, private schools in Chandigarh reserve some places for more disadvantaged children, allowing them to benefit from the better facilities at these schools.

There has been substantial lobbying at the territory level to roll out this accreditation scheme state- and ultimately country-wide. As a result, a project is already in preparation to accredit all schools of the Union Territory of Chandigarh and Hyderabad city in Andhra Pradesh. As part of this project, there is also provision for a cross-sectional study to assess the health profiles of students in HPS, to compare students from higher (platinum/gold) and lower (bronze) HPS, to examine the effect of accreditation at the individual level.

It is recommended that the developed accreditation manual and checklist be used in all school settings in Chandigarh and can be piloted in other parts of India. This will help in providing feedback on the implementation of this accreditation tool in India. An intervention study carried out in Chandigarh in 2008 concluded that the health promotion model in school settings is feasible and must be integrated into the school education system. It was suggested that physical activity and dietary guidelines should also be part of the curriculum, for which a change at policy level is required [17].

## Conclusions

The HPS accreditation system is feasible in school settings and was well implemented in the schools of Chandigarh. Improvements in accreditation scores between 2011 and 2013 suggest that the system may be effective in increasing levels of health promotion in communities.
